# A lonely electron blocks incoming pairs

**DOI:** 10.1016/j.jbc.2021.100294

**Published:** 2021-02-12

**Authors:** Marta Massari, Callum R. Nicoll, Andrea Mattevi

**Affiliations:** Department of Biology and Biotechnology “Lazzaro Spallanzani”, University of Pavia, Pavia, Italy

**Keywords:** flavin, electron bifurcation, flavoprotein, enzyme mechanism

## Abstract

Electron bifurcation exploits high energetic states to drive unfavorable single electron reactions and determining the overall mechanism governing these electron transfers represents an arduous task. Using extensive stopped-flow spectroscopy and kinetic simulations, Sucharitakul *et al.* now explore the bifurcation mechanism of the electron transfer flavoprotein EtfAB from the anaerobic gut bacterium *Acidaminococcus fermentans*. Strikingly, they illustrated that catalysis is orchestrated by a negatively charged radical, α-FAD, that inhibits further reductions and features an atypical inverted kinetic isotope effect. These results provide additional insight behind electron transfers that are prevalent within multienzyme governed reactions.

Electron bifurcation, in which a two-electron donor sends its electrons to two distinct acceptors, was first discovered in the Q-cycle of complex III of the electron transport chain (1). Bifurcation couples endergonic and exergonic reactions together. The energy demand for the unfavored (endergonic) reaction is compensated by the energy gained through the highly favorable (exergonic) reaction (1). More recently, electron bifurcation performed by flavins has been observed in several anaerobic bacteria and *Archea* (2, 3, 4, 5). In flavin-based electron bifurcation, the cofactor NAD(P)H reduces the flavin adenine dinucleotide (FAD) to the hydroquinone state, FADH^−^. FADH^−^ bifurcates one electron to an acceptor with a low reduction potential (unfavored reaction) and one electron to an acceptor with a high reduction potential (favored), which compensates for the former energetic cost (4). For the bifurcating electron transfer flavoprotein from *Acidaminococcus fermentans*, EtfAB, the initial electron acceptor is an FAD in the B-subunit (β-FAD), while a neighboring FAD in the A-subunit (α-FAD) serves as the “uphill” (ΔΔV= −208 mV) electron destination. Specifically, in the catalytic reaction, NADH first reduces the hydride acceptor and bifurcating β-FAD (Fig. 1*A*). The two-electron reduced β-FADH^−^ then transfers one electron to the semiquinone state of α-FAD (α-FAD^•−^), to form the hydroquinone (α-FADH^−^). This electron transfer is allowed due to a rotation of the α-FAD-containing domain (6) (Fig. 1*B*). The other electron is bifurcated to a [4Fe-4S] cluster of a ferredoxin molecule. A further rotation of the α-FAD-containing domain allows one electron transfer from α-FADH^−^ to δ-FAD of the butyryl-CoA dehydrogenase (6, 7) (Fig. 1*C*). The elucidation of these steps certainly explains the path each electron takes, but the mechanistic details of these processes remain unclear. New insights into this mechanism not only expand our enzymatic understanding but also can be applied to facilitate new chemical reactions or energetic transformations.

**Figure 1:**
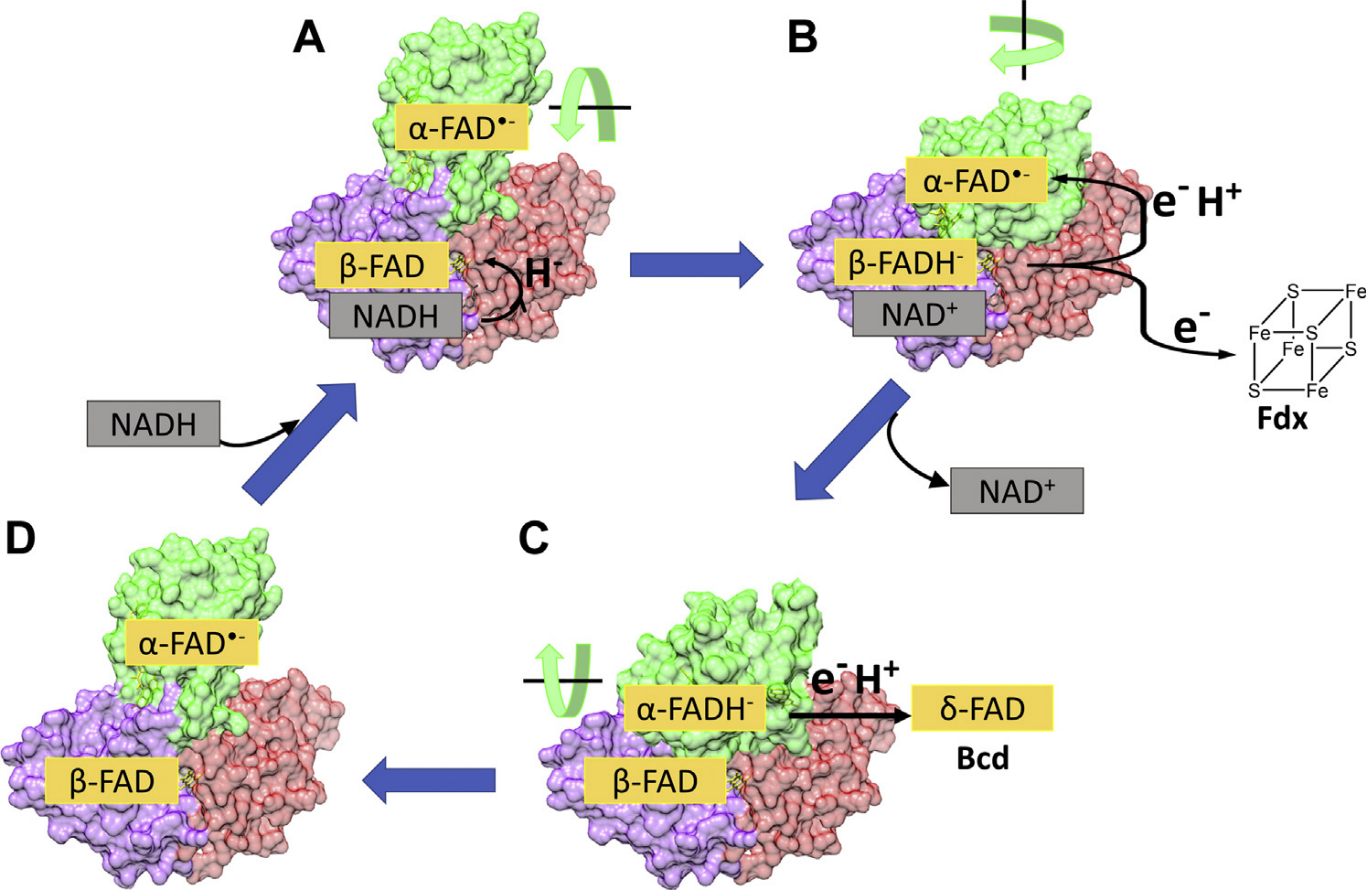
**Representation of EtfAB electron bifurcation mechanism (PDB: 4L2I).***A*, NADH reduces β-FAD to β-FADH^−^. *B*, the hydroquinone form of β-FAD bifurcates one electron to α-FAD^•−^ that is reduced to α-FADH^−^ and one electron to the [4Fe-4S] cluster of a ferredoxin (Fdx). In the absence of the ferredoxin, the electron is bifurcated to the α-FAD of a second EtfAB molecule. *C,* α-FADH^−^ rotates toward δ-FAD of butyryl-CoA dehydrogenase (Bcd) that is in complex with EtfAB, forming δ-FADH^•^ and α-FAD^•−^, and *D,* rotates back. A second round of bifurcation reduces another ferredoxin molecule and α-FAD^•−^ to α-FADH^−^, leading again to domain rotation and reduction of δ-FADH^•^ to δ-FADH^−^. At this point, Bcd can reduce crotonyl-CoA to butyryl-CoA (6).

To explore this process, Sucharitakul *et al.* (8) returned to EtfAB from *A. fermentans* as a test case. The authors performed extensive stopped-flow kinetics to rationalize the bifurcating mechanism underlining catalysis. They began by first analyzing the principal reduction step governed by NADH and β-FAD. The step involves two phases, fast and slow, that represent the formation of the charge-transfer complex, β-FADH^−^:NAD^+^ (fast), followed by its decay and electron transfer to α-FAD^•−^ (slow). Intriguingly, reducing α-FAD to form the negatively charged semiquinone radical, α-FAD^•^^−^, took place both *intra*- and *inter*molecularly, in the absence of ferredoxin. In other words, β-FADH^−^ reaches out to additional molecules of EtfAB to fulfill the bifurcation process for both electrons when a physiological acceptor is not available. A further experiment in the presence of butyryl-CoA dehydrogenase and ferredoxin showed a turnover number of EtfAB comparable with that determined in a kinetic simulation of the reduction of β-FAD in presence of α-FAD^•−^, confirming that the α-FADH^−^ form is responsible for the electron transfer to butyryl-CoA dehydrogenase (Fig. 1).

Sucharitakul *et al.* (8) further noted a striking redox-state dependent cross talk between the two flavins. The initial phase of the β-FAD reduction was enhanced in the presence of α-FAD (compared to EtfaB, without α-FAD). By contrast, the rate of reduction for the β-FAD molecule was stalled by the presence of the α-FAD^•−^ semiquinone, by approximately 1500-fold. Sucharitakul *et al.* further investigated the fast phase of the β-FAD reduction by measuring kinetic isotope effects (KIEs). By using deuterated NADH (NADD), the authors determined a KIE of 2.1 for the β-FAD reduction in the presence of the oxidized α-FAD. Moreover, the KIE was only detectable for the reduction step and not the interflavin electron transfer. This effect was not surprising because the larger atomic weight of the deuterium is generally expected to slow down the reaction rates. The surprise came when the authors measured the KIE for the β-FAD reduction in the presence of α-FAD^•−^. While it was confirmed that α-FAD^•−^ restricts the rate of reduction overall, NADD reduced β-FAD more quickly than NADH. This inverted KIE is definitively unusual, indicating that electron bifurcation must be governed by unique rules.

In light of such a puzzling behavior, Sucharitakul *et al.* sought to verify whether the radical, or the negative charge, component of the α-FAD^•−^ semiquinone thwarted β-FAD reduction. Since α-FAD possesses a higher two-electron reduction potential than β-FAD, they were able to prepare “half-reduced” EftAB, possessing β-FAD, and the fully reduced α-FADH^−^, using dithionite-based titrations. They showed that the rate of reduction was similar to that observed with oxidized α-FAD bound. Additionally, the rates were equivalent regardless of whether dithionite or excess NADH was used to generate α-FADH^−^. Therefore, the authors unequivocally demonstrated that the inherent characteristics of the radical, not the charge, of the α-FAD^•−^ semiquinone impede the reduction of β-FAD.

This study provides a striking example of the redox chemistry of a bifurcating electron-transfer flavoprotein, in which the radical form of the downstream flavin (the α-FAD) modulates the reactivity of the upstream flavin (the β-FAD). It would not seem that it is a long-range electrostatic effect because the negatively charged fully reduced α-FADH^−^ does not exert this effect. As usual, good work raises more questions than answers. What is the molecular and electronic mechanism underlying such an effect? Is the radical state affecting the domain orientation? If yes, how? Or is it a matter of the protein matrix functioning as an electron conduit that functionally couples the redox and kinetic properties of the two flavins when one of them is in the radical state? Is the observed intermolecular electron transfer between EftAB proteins physiologically relevant? The excellent manuscript by Sucharitakul *et al.* (8) will pave the way to more work for the disclosure of the mechanism of other “bifurcating” flavoenzymes.

## Conflict of interest

The authors declare that they have no conflicts of interest with the contents of this article.
